# Characteristics and outcome of children with renal tumors in the Netherlands: The first five-year’s experience of national centralization

**DOI:** 10.1371/journal.pone.0261729

**Published:** 2022-01-13

**Authors:** Prakriti Roy, Sophie E. van Peer, Martin M. de Witte, Godelieve A. M. Tytgat, Henrike E. Karim-Kos, Martine van Grotel, Cees P. van de Ven, Annelies M. C. Mavinkurve-Groothuis, Johannes H. M. Merks, Roland P. Kuiper, Janna A. Hol, Geert O. R. Janssens, Ronald R. de Krijger, Marjolijn C. J. Jongmans, Jarno Drost, Alida F. W. van der Steeg, Annemieke S. Littooij, Marc H. W. A. Wijnen, Harm van Tinteren, Marry M. van den Heuvel-Eibrink

**Affiliations:** 1 Princess Máxima Center for Pediatric Oncology, Utrecht, The Netherlands; 2 Department of Research and Development, Netherlands Comprehensive Cancer Organization (IKNL), Utrecht, The Netherlands; 3 Department of Clinical Genetics, University Medical Center Utrecht (UMCU), Utrecht, The Netherlands; 4 Department of Radiation Oncology, University Medical Center Utrecht (UMCU), Utrecht, The Netherlands; 5 Department of Pathology, University Medical Center Utrecht (UMCU), Utrecht, The Netherlands; 6 Oncode Institute, Utrecht, The Netherlands; 7 Department of Radiology and Nuclear Medicine, University Medical Center Utrecht/Wilhelmina Children’s Hospital (UMCU), Utrecht, The Netherlands; Cincinnati Children’s Hospital Medical Center, UNITED STATES

## Abstract

Around 6% of all childhood malignancies represent renal tumors, of which a majority includes Wilms tumor (WT). Although survival rates have improved over the last decades, specific patients are still at risk for adverse outcome. In the Netherlands, since 2015, pediatric oncology care for renal tumors has been centralized in the Princess Máxima Center for Pediatric Oncology. Here, we describe experiences of the first 5 years of centralized care and explore whether this influences the epidemiological landscape by comparing data with the Netherlands Cancer Registry (NCR). We identified all patients <19 years with a renal mass diagnosed between 01-01-2015 and 31-12-2019 in the Princess Máxima Center. Epidemiology, characteristics and management were analyzed. We identified 164 patients (including 1 patient who refused consent for registration), in our center with a suspicion of a renal tumor. The remaining 163 cases included WT (n = 118)/cystic partially differentiated nephroblastoma (n = 2)/nephrogenic rests only (n = 6) and non-WT (n = 37). In this period, the NCR included 138 children, 1 17-year-old patient was not referred to the Princess Máxima Center. Central radiology review (before starting treatment) was performed in 121/163 patients, and central pathology review in 148/152 patients that underwent surgery. Treatment stratification, according to SIOP/EpSSG protocols was pursued based on multidisciplinary consensus. Preoperative chemotherapy was administered in 133 patients, whereas 19 patients underwent upfront surgery. Surgery was performed in 152 patients, and from 133 biomaterial was stored. Centralization of care for children with renal tumors led to referral of all but 1 new renal tumor cases in the Netherlands, and leads to referral of very rare subtypes not registered in the NCR, that benefit from high quality diagnostics and multidisciplinary decision making. National centralization of care led to enhanced development of molecular diagnostics and other innovation-based treatments for the future.

## Introduction

Around 6% of all childhood malignancies represent kidney tumors [1, 2]. The majority of these patients suffer from nephroblastoma, or Wilms tumor (WT), while around 10–15% are suggested to be diagnosed with a variety of other tumors, the so-called ‘non-WTs’ [3, 4]. Mesoblastic nephroma (MN) and malignant rhabdoid tumor of the kidney (MRTK) present predominantly in infants [5–10], whereas renal cell carcinoma (RCC) is the most common malignancy in children over the age of 14 years [5, 11–13]. Clear cell sarcomas of the kidney (CCSKs) occur at the same age as WTs (median age 2–3 years) [14–16]. The exact prevalence of certain entities such as MN, Cystic nephroma (CN) and angiomyolipoma is unknown, as they are not systematically registered in cancer registries.

Risk stratification of pediatric kidney tumors for treatment is mainly based on stage and histological subtype [2, 5, 17]. The histological classification and staging procedures of kidney tumors in The Netherlands is based on the strategy of the International Society of Pediatric Oncology Renal Tumor Study Group (SIOP-RTSG) which advocates preoperative chemotherapy treatment [2]. Except for diffuse anaplastic and blastemal type WT cases, overall survival rates are excellent (~90%) for unilateral WTs and evidence-based treatment reduction strategies are already being pursued over the past 2 decades [18, 19]. Cystic partially differentiated nephroblastoma (CPDN) is considered a low risk nephroblastoma with excellent survival [20]. Similar high survival rates apply to, amongst others, MN, (mostly *DICER1* driven) CN, angiomyolipomas and metanephric (fibro-)adenomas which have excellent outcomes, treated by surgery only [2, 20, 21]. However, there are still some challenges in the renal tumor field. Patients with localized RCC have particularly high overall survival rates, however when presenting with distant metastases, outcome is very poor [22–25]. MRTKs characterized by (*SMARCB1* (95%) and *SMARCA4* (5%) aberrations [26]) represent a highly chemotherapy-insensitive tumor with a typically poor outcome. It represents one of the greatest challenges for the molecular based novel treatment development in the pediatric renal tumor field on an international level [27–29]. Moreover, clinical challenges include the management of relapsed CCSKs, high risk histology WTs and bilateral tumors [15, 30–36].

Centralized care for patients suffering from pediatric malignancies has been hypothesized to enhance cure rates. Research has already proven that higher volume hospitals present higher overall survival rates for a broad range of tumor types, without any negative effects [37, 38]. A multidisciplinary approach by experts in the field of diagnostics, stratification and therapy is of utmost importance for children with renal tumors [5]. Especially, skills to review diagnostics, multidisciplinary decision making and development of innovations in diagnostic procedures and therapeutic strategies are important to decrease morbidity, mortality and to improve long term outcomes [39]. In addition, it is conceivable that referral and registration of all pediatric renal tumor patients in 1 national center may give a more accurate insight in the epidemiology of tumor subtype distribution. This may even identify previously disguised rare subtypes of renal tumors that may benefit from multidisciplinary management.

With the mission to optimize outcome and to reduce early and late toxicity for children with cancer, the Princess Máxima Center for Pediatric Oncology was founded in November 2014 in Utrecht, the Netherlands. Here, we describe the epidemiology and experiences, including referral patterns, of the first 5 years of national centralization of care for all children with a newly diagnosed renal tumor in the Princess Máxima Center for Pediatric Oncology in the Netherlands as compared to registration data in the Netherlands Cancer Registry (NCR).

## Methods

### Patients

In this descriptive report, all patients presenting with a renal mass in the Princess Máxima Center for Pediatric Oncology between January 1^st^ 2015 and December 31^st^ 2019 were included. All included patients and/or parents, provided written informed consent for registration (EudraCT numbers 2007-004591-39, 2016-004180-39, 2005-001139-31, with ethics committee approval numbers MEC 202.134/2001/122, MEC-2018-026, MEC-2006-348 respectively and Netherlands Trial Register NL7744 with ethics committee approval number MEC-2016-739). Data including sex, age at diagnosis, presenting symptoms, radiological and pathological (review) classifications, surgery (timing and procedure), biobanking, pre- and postoperative treatment and follow-up data, were retrieved from the medical records and patient registry of the Princess Máxima Center.

### Diagnostics, treatment and biobanking

Stage and histology were classified according to the SIOP-RTSG histological classifications [5, 40]. Standard diagnostic radiology advice consisted of abdominal ultrasound and chest X-ray in the SIOP 2001 protocol which were used until June 2019. However, from 2015 onwards, abdominal magnetic resonance imaging including diffusion weighted imaging (MRI-DWI) as well as abdominal ultrasound and chest computed tomography (CT), as advised in SIOP-RTSG 2016 UMBRELLA, were implemented whenever feasible, in our setting. Central radiology and pathology review were performed by members of the international SIOP-RTSG review panels, after local assessment, and are considered standard since June 2019 [17, 40]. Treatment was based on recommendations according to the SIOP 2001 and the SIOP-RTSG 2016 UMBRELLA protocols, and in these years for MRTKs, the EpSSG-NRSTS 2005 treatment protocol was used.

After obtaining informed consent, tumor tissue, normal kidney tissue, urine and peripheral blood from individual patients and parents was biobanked. This biobanking procedure was according to SIOP protocols and from 2018 onwards, also according to the standard biobanking procedure of the Princess Máxima Center (MEC-2016-739, Netherlands Trial Register (NTR) NL7744).

### Comparison with Netherlands Cancer Registry (NCR)

We compared the registry of patients younger than 19 years of age, presenting with a renal mass in the Princess Máxima Center, with renal tumor patients registered in the NCR database during the study period, The NCR uses the ICD-O-3 coding of disease classification and is based on notification of all newly diagnosed malignancies in The Netherlands by the Nationwide Network and Registry of Histopathology and Cytopathology (PALGA) and the National Registry of Hospital Discharges [41].

## Results

### Clinical characteristics at presentation

**Fig 1:**
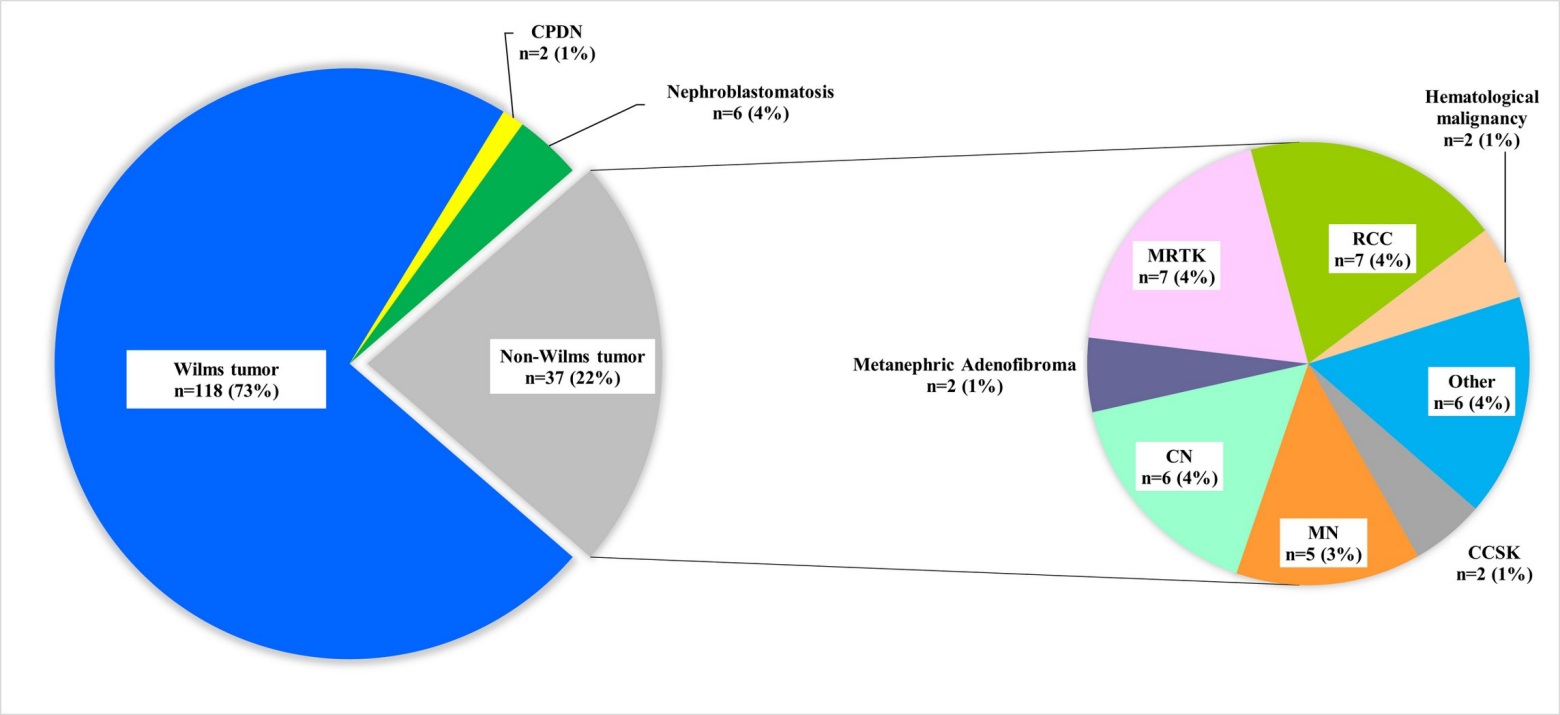
Distribution of renal cancer subtypes in patients with suspicion of renal tumor. RCC: renal cell carcinoma, CCSK: clear cell sarcoma of the kidney, MN: mesoblastic nephroma, CN: cystic nephroma, MRTK: malignant rhabdoid tumor of the kidney.

**Fig 2:**
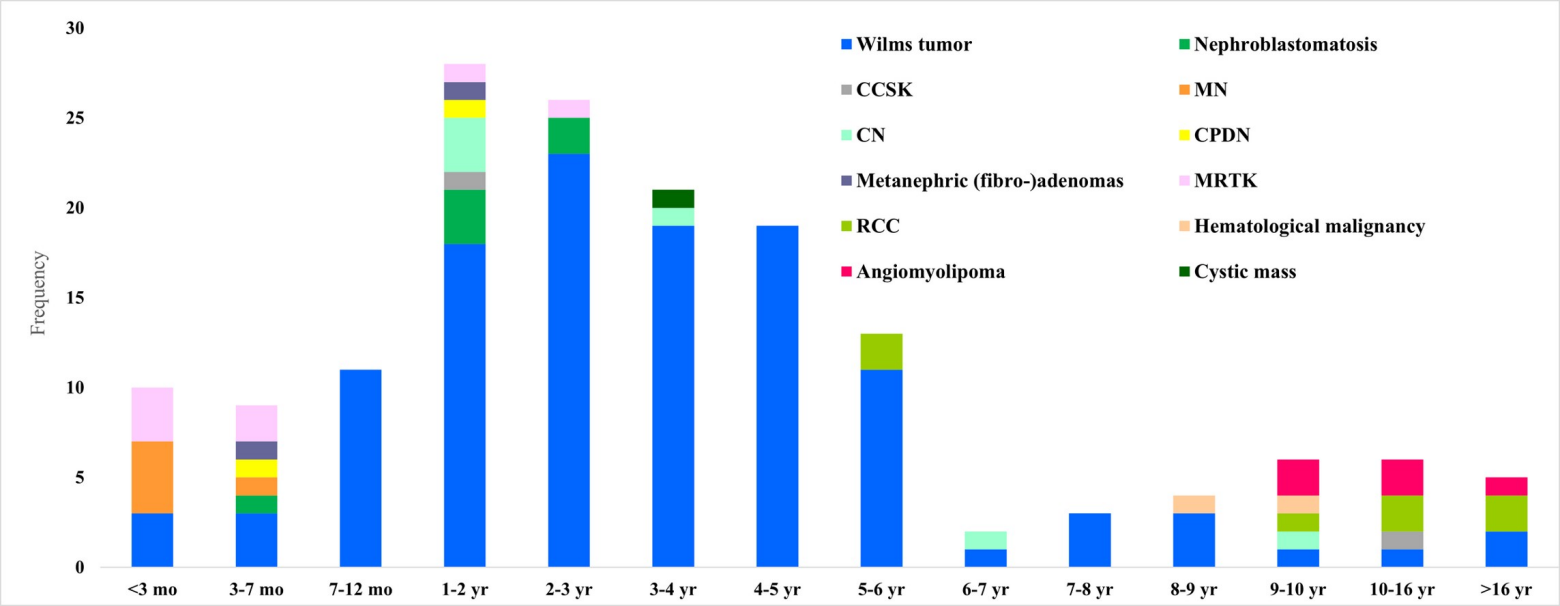
Age distribution of registered patients per tumor sub-types. CCSK: clear cell sarcoma of the kidney, CN: cystic nephroma, RCC: renal cell carcinoma, MN: congenital mesoblastic nephroma, CPDN: cystic partially differentiated nephroblastoma, MRTK: malignant rhabdoid tumor of the kidney; mo: months, yr: years.

Between January 1^st^ 2015 and 31^st^ December 2019, 164 new patients (76 male (46%), 88 female (54%)) with a renal mass were referred to the Princess Máxima Center. The parents of 1 patient refused to register data and this patient was therefore excluded from this report. The median age at presentation of the remaining 163 patients was 35 months (range: 0–226 months) (Table 1). The distribution of renal tumor subtypes (WT/CPDN, nephrogenic rests, CCSK, MRTK, CN, MN, RCC, angiomyolipoma and others) are listed in Table 1 and Fig 1. Sixty-four (39%) patients presented with a prominent abdominal mass and 48 (29%) with abdominal pain. Asymptomatic presentation was recorded in 29 (15%) patients. In these asymptomatic patients, the renal tumor was identified either by screening for renal tumors in patients with a known genetic predisposition (n = 11) or as an incidental finding on imaging for trauma, urine tract infections or other indications (n = 18). WT patients presented at a median age of 35 months (range 0–226 months), including 2 patients who were 18 years at diagnosis (Fig 2).

**Table 1:** Characteristics overview of registered patients with suspicion for a renal tumor, 2015–2019.

Disease	n	Median age in months (range)	Stage^1^									Subtype						Protocol		Mortality		Recurrence		
			Localized			Metastasized					NA							SIOP 2001	SIOP-RTSG 2016 UMBRELLA	DRM	TRM	LR	IR	HR
			I	II	III	V	I	II	III	V														
WT	118	37 (0–226)	30	24	21	16	5	4	18	4	0	**LR**	**IR**	**HR-DA**		**HR-BT**		106	12	1	2	0	5	1
												2	99	7		10								
CPDN	2	12 (6–18)	1	0	1	0	0	0	0	0	0	2	0	0		0		2	0	0	0	0		
NB/NR only	6	16 (6–25)	Unilateral: 3									**ILNR**		**PLNR**		**NA**		5	1	0	0	0		
			Bilateral: 3									2		1		3								
MRTK*	7	3 (0–28)	0	2	0	0	0	0	3	0	2	NA						7	0	5	0	0		
CCSK	2	70.5 (19–122)	1	1	0	0	0	0	0	0	0	NA						2	0	0	0	0		
RCC	8	179 (63–196)	3	1	2	1	0	1	0	0	0	**T**	**C**	**P**	**P+S**	**FH**	**NOS**	5	2	1	0	0		
												2	1	1	1	1	2							
CN	5	20 (13–116)	0	0	0	0	0	0	0	0	5	NA						5	1	0	0	0		
MN	5	0 (0–6)	0	4	1	0	0	0	0	0	0	**Classic**		**Mixed**		**Cellular**		4	1	0	0	0		
												2		2		1								
NHL	1	111	NA									NA						0	1	0	0	0		
ALL	1	97	NA									NA						1	0	0	0	0		
Metanephric (fibro-) adenoma	2	14 (6–21)	NA									NA						2	0	0	0	0		
Angiomyolipoma	5	126 (110–198)	NA									n = 5 confirmed tuberous sclerosis						5	0	0	0	0		
Benign cyst	1	40	NA									NA						1	0	0	0	0		
**Total**	163	35 (0–226)	35	32	25	20	5	5	21	4	7	-						145	18	7	2	5		

n: number of patients.

NCR: Netherlands Cancer Registry.

NA: not available.

SIOP: International Society of Pediatric Oncology.

RTSG: Renal Rumor Study Group.

DRM: disease related mortality.

TRM: treatment related mortality.

LR: low risk.

IR: intermediate risk.

HR: high risk.

WT: Wilms tumor.

HR-DA: high risk diffuse anaplastic Wilms tumor.

HR-BT: high risk blastemal type Wilms tumor.

NR/NB: nephrogenic rest/nephroblastomatosis.

ILNR: intralobar nephrogenic rests.

PLNR: perilobar nephrogenic rests.

CPDN: cystic partially differentiated nephroblastoma.

MRTK: malignant rhabdoid tumor of the kidney.

RCC: renal cell carcinoma.

T: translocation type.

C: clear cell type RCC.

P: papillary type RCC.

P+S: papillary type RCC with sarcomatoid components.

FH: *FH*-mutation related RCC.

NOS: not otherwise specified.

CN: cystic nephroma.

MN: congenital mesoblastic nephroma.

CCSK: clear cell sarcoma of the kidney.

NHL: non-Hodgkin lymphoma.

ALL: acute lymphoblastic leukemia.

^1^Stage according to SIOP 2001 and SIOP-RTSG 2016 UMBRELLA classification [5, 17].

*Registered in SIOP-RTSG for diagnostics and biobanking but treated according to EpSSG protocols.

### Comparison with the Netherlands Cancer Registry

In the same period, the NCR registered 138 patients with renal tumors aged 0–18 years. These included WT (n = 117), RCC (n = 9), MRTK (n = 7), CCSK (n = 1), angiomyolipoma (n = 1), and B-cell non-Hodgkin lymphoma (B-NHL) (n = 1) (Table 2). Twenty-five referred patients with MN, CN, nephroblastomatosis, angiomyolipomas, cystic masses and metanephric (fibro-)adenomas had not been systematically registered in the NCR (Table 2).

**Table 2:** Patients presenting with a renal mass in the Princess Máxima Center compared with patients registered in the National Cancer Registry.

Renal mass	Registration in Princess Máxima Center	Registration in NCR
**WT**	118	117
**CPDN**	2	2
**NB/NR only**	6	0
**MRTK**	7	7
**CCSK**	2	1
**RCC**	8	9
**CN**	5	0
**MN**	5	0
**NHL**	1	1
**ALL**	1	0
**Metanephric (fibro-) adenoma**	2	0
**Angiomyolipoma (TS)**	5	1
**Cystic mass**	1	0
**Total**	**163**	**138**

NCR: Netherlands Cancer Registry.

WT: Wilms tumor.

CPDN: Cystic partially differentiated nephroblastoma.

NB: nephroblastomatosis.

NR: nephrogenic rest.

MRTK: malignant rhabdoid tumor of the kidney.

CCSK: clear cell sarcoma of the kidney.

RCC: renal cell carcinoma.

CN: cystic nephroma.

MN: (congenital) mesoblastic nephroma.

NHL: non-Hodgkin lymphoma.

ALL: acute lymphoblastic leukemia.

### Diagnostic procedures

In 149/163 children (91%) a diagnostic abdominal MRI-DWI, in 6/163 children (4%) an abdominal CT-scan and in 8/163 patients (all diagnosed before 2017) an abdominal ultrasound only, had been performed at diagnosis. Of the 137 patients with WT, CPDN, CCSK, MRTK and RCC, 122 (89%) underwent chest CT, and 14 had chest X-ray only (mostly before 2017) in search of pulmonary metastases. As standard of diagnostic care, prior to the start of treatment, central radiology review had been performed in 121 of 163 (74.2%) patients, (105/145 included in SIOP 2001 and 16/18 included in SIOP-RTSG 2016 UMBRELLA), by a SIOP-RTSG panel radiologist. After the initiation of the SIOP-RTSG 2016 UMBRELLA protocol in June 2019, all but 1 patients underwent MRI-DWI and chest CT-scan.

Fine needle biopsies before starting treatment were performed in 20 cases. Eight of these biopsies confirmed a diagnosis of WT, after which preoperative chemotherapy was started. Reasons for biopsy in those 8 cases were higher age at diagnosis (n = 2), high serum alpha fetoprotein (n = 2), atypical presentation on imaging (n = 2), atypical presentation with respiratory insufficiency due to a pulmonary tumor mass (n = 1) and elevated serum uric acid and lactate dehydrogenase (n = 1). The other 12 biopsies revealed angiomyolipomas (n = 3), RCC (n = 2), MRTK (n = 2), CCSK (n = 1), B-NHL (n = 1), ALL (n = 1), MN (n = 1) and nephroblastomatosis (n = 1).

### Disease characteristics

In total, 139/163 patients presented with unilateral disease and 24/163 presented with bilateral disease. Metastases were diagnosed in 33 (24%) of 137 patients with a malignant renal tumor, of which 4 had bilateral disease. Five WT patients presented with a tumor thrombus into the inferior vena cava, of which 2 with extension into the heart.

### Treatment

**Fig 3:**
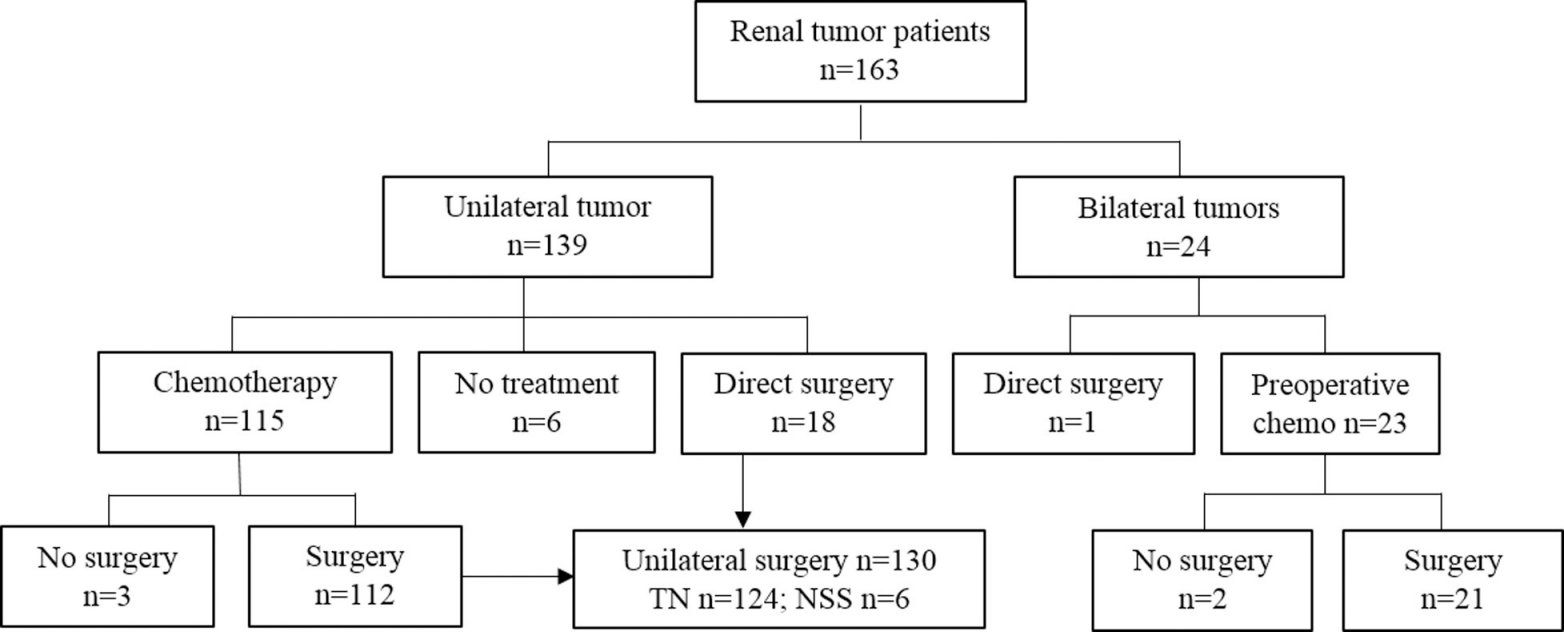
Flow diagram of presentation and surgical management of patients. n: number of patients; chemo: chemotherapy; TN: tumornephrectomy; NSS: nephron sparing surgery.

Surgery was performed in 152 patients and preoperative chemotherapy was administered in 133/152. In 19 patients an upfront surgery was performed, because of age below 7 months (n = 8), radiological suspicion of cystic nephroma or other cystic diseases (n = 6), or a suspicion, or (histological) confirmation, of RCC (n = 5). RCC was suspected based on higher age, genetic predisposition (germline fumarate hydratase (*FH)*-mutation) or radiological characteristics. Of the 130 unilateral cases that underwent surgery, 124 (95%) underwent complete tumor nephrectomy and 6 (5%) patients underwent partial nephrectomy (Fig 3). Details of management and outcome of the bilateral cases will be reported separately.

As mentioned above, 11 patients did not undergo surgery. Six of these 11 patients did not receive any chemotherapy, and did not have a resection of the renal mass due to suspicion of stable renal non-malignant lesions. Among them, 3 patients had small, but increasing in size, biopsy-confirmed angiomyolipomas (all with confirmed tuberous sclerosis) and 2 patients had small CNs. These 2 CN patients were closely monitored using MRI-DWI and revealed stable size renal masses after 3 years.

Five of the 11 patients without resection did receive chemotherapy, due to a strong suspicion (or confirmation) of bilateral nephroblastomatosis (n = 2, 1 after biopsy), or biopsy confirmed hematological malignancy (having normal blood counts, 1 ALL and 1 B-NHL, n = 2). One more patient who received only chemotherapy, but no surgery, was a patient with a confirmed diagnosis of extensive MRTK (based on biopsy) who deceased during preoperative chemotherapy. Another 1-month old infant with biopsy-confirmed MRTK with a germline *SMARCB1-*deletion had a concomitant large atypical teratoid rhabdoid tumor of the brain at presentation. Shared decision making with the parents resulted in waiving any treatment and starting palliative care.

One patient in a family with a known germline *FH*-mutation, presented with a benign cystic renal mass in 2017 based on radiology and was therefore only observed (without surgery or chemotherapy). However, close wait and watch surveillance of the lesion revealed change of diffusion restriction pattern on MRI-DWI in 2021, based on which it was decided to completely remove the lesion (complete resection by partial nephrectomy). Histology of this lesion turned out to be an *FH*-related RCC. This patient is currently in now in complete remission.

### Histology

Stage of all renal tumor patients is summarized in Table 1. In 148/152 patients, after surgery, histological review has been performed by a SIOP-RTSG review pathologist [17, 40]. From the start of the SIOP-RTSG 2016 UMBRELLA protocol, in June 2019, all patients were stratified for postoperative treatment, based on centralized rapid pathology review (CPR).

Of 133/152 patients who underwent surgery, biomaterial could be stored upon retrieval of SIOP-RTSG and Princess Máxima Center biobank written informed consent. Reasons for not storing material from June 2019 on was mainly retrieval of necrotic tumor, without solid components.

### Postoperative management

Apart from chemotherapy, 54 patients required radiotherapy (flank n = 33, whole abdomen n = 5, combination of whole abdomen/flank with lung n = 7, whole lung only n = 6 and others n = 3). All of these were treated with image guided, intensity-modulated radiation therapy (IMRT) techniques as recently described [42, 43].

#### WT and CPDN

With a median follow-up time of 42 months (3.5 years, range 1–73 months), 6 WT patients experienced a relapse (initial histology: n = 1 blastemal type, n = 5 intermediate risk histology) and survived after relapse treatment [2].

In 1 of the metastasized WT patients with regressive WT, a concomitant embryonal rhabdomyosarcoma (RMS) in the brain was identified, which became apparent during therapy. This patient died due to complications of progression of the cerebral RMS despite intensive treatment. Two other (diffuse anaplastic (DA) bilateral WT) patients died due to extensive treatment-related toxicity, i.e. Candida infection/necrotic intestine and circulatory shock, respectively. Both stage V patients had compromised kidney function, but that was not the primary cause of death. Both had developed of kidney failure 3- and 7-months post-surgery, respectively.

#### MRTK

The localized MRTK patients (n = 2) underwent chemotherapy, surgery and radiotherapy. Currently, they are alive and well with a follow-up time of 38 and 67 months respectively. Five MRTK patients presented with metastatic disease, 4 of these were therefore intensively treated, and in 1 treatment was waived, based on shared decision making (see aforementioned details). All metastatic MRTK patients ultimately died.

#### RCC

Among the 8 RCC patients with a median follow-up of 47 months (range: 13–56 months), 1 patient with metastatic papillary type RCC (TFE negative) with sarcomatoid components experienced disease progression. Despite treatment with sunitinib and second-line axinitinib, this patient died. The other patients included translocation type RCC (n = 2), clear cell type RCC (n = 1), and RCC not otherwise specified (NOS) (n = 2), all presented with localized disease. Two RCC patients with germline *FH*-mutations were of the *FH*-related subtype. These RCCs were surgically removed and the patients are in CCR, with a median follow-up time of 47 months (range 13–56 months) [44].

#### CCSK

The 2 CCSK patients (stage I and II) were treated according to the SIOP-RTSG 2016 UMBRELLA protocol and are alive and well at a follow-up period of 50 and 72 months.

#### MN

The 5 MN patients (classic type n = 2, mixed type n = 2 and cellular type = 1) presented localized disease (stage II and III respectively), were treated with surgery only, and are alive and well with a median follow-up of 29 months (range 16–68 months). The cellular type MN had an *ETV6-NTRK3* translocation.

All angiomyolipoma, CN, CPDN, renal cystic masses, and metanephric (fibro-)adenoma (n = 2) patients are in complete remission and alive and well at last follow-up, after a median follow-up period of median 43 months (range 0–73 months).

## Discussion

The mission of the ambitious effort of centralizing pediatric oncology care in the Netherlands, is to eventually cure every child with cancer, with optimal quality of survival. This brought experts and scientists together, which led to the infrastructure of standardized diagnostics (with central review), management and registration of all pediatric cancers. This initiative was reinforced by studies that claimed better outcome in patients who were treated in specialized and high-volume settings [37, 38, 44]. The management of pediatric renal tumors on a national level was taken forward from the very beginning of our hospital in November 2014. When we compare our registration data with the national cancer registry in The Netherlands (NCR database), it demonstrates that we indeed managed to attract all but 1 Dutch patients with renal tumors that are considered ‘malignant’ (WT/CPDN, CCSK, MRTK, RCC) until the age of 19 years from the very first moment. In total, 37 (22%) patients were not WT/CPDN/nephroblastomatosis/nephrogenic rest (non-WT), which is higher than non-WT percentages in reported series [45, 46]. This illustrates that centralization of care leads to a more complete epidemiological picture of renal tumor subtypes. Cancer registries, using the ICD-O-3 classification and the ICCC-3 coding, are often not including these renal tumor subtypes as separate entities. Renal masses that represent MN, angiomyolipomas, CN and metanephric (fibro-)adenoma are however important entities within the pediatric oncology field, that benefit from multidisciplinary decision making after high end reviewed diagnostics. In addition, by including these diseases in the pediatric oncology spectrum, novel molecular characteristics can be identified that may lead to individual targeted treatment approaches in cases that are considered rather benign, but start to behave aggressively. Some of such rare entities have already shown to be strongly associated with specific genetic drivers, or proven to be part of predisposition syndromes (tuberous sclerosis related angiomyolipomas and *FH*-related RCCs) [44]. Moreover, some of these non-ICCC-3 coded entities can carry molecular aberrations driving clinical aggressiveness in specific cases (for example MN or CN) [7, 20]. Therefore, it is important that these rare cases are referred to pediatric oncology centers and are registered as separate entities. This is now being reinforced in SIOP-RTSG 2016 UMBRELLA, with the aim to gather information that may inform future decision making [2].

Of the 3 17- and 18-year-old patients in the NCR database, only 1 was not referred to our center. This reflects the dilemma of referral in teenagers and young adults. We acknowledge the importance of collaboration with adult and young adolescent (AYA) oncologists, and take this aim forward for the future in order to provide the best oncological and psychological care for AYA patients. This is especially important, as the molecular spectrum of these diseases and consequently clinical behavior and optimal therapy choice may be different for adults compared to children (as shown in RCC). Fortunately, the 2 18-year-old Wilms tumor patients that were referred to our hospital, did not suffer from any treatment delay, which has often been reported in WT patients in adult oncology settings, and is a determinant of adverse outcome [47].

MN patients often present prenatally or as newborns, and a high risk of surgery-related morbidity and mortality has been reported in such infants [7]. Moreover, it has recently been shown that a subset of MNs can behave aggressively [48]. This stresses the importance, despite the relative benign character of most cases, to refer these infants to national renal tumors expert centers. In these centers, it is possible to provide them with well-equipped diagnostics, multidisciplinary decision making and tertiary surgery expertise and to avoid unnecessary morbidity and mortality.

Radiology review has become standard of care according to the current SIOP-RTSG 2016 UMBRELLA protocol for treatment stratification (2). This expert review is important, for instance to distinguish metastases from benign lung lesions such as intra-pulmonary lymph nodes [49]. On the other hand, metastases can be overlooked. Over time, the use of MRI-DWI and the logistic settings to use these radiology modalities for all patients (including young patients that require anesthesia) became available for diagnostic as well as for response assessment purposes in our settings [50]. It is an important goal for the near future to enhance discrimination of renal tumor subtypes by radiological prediction of histological subtypes based on MRI-DWI characteristics. This is an effort which is embedded in the international aims of the SIOP-RTSG 2016 UMBRELLA protocol [2], and also includes nephrometry scoring in collaboration with the COG-RTG within the framework of the HARMONICA initiative [51].

The international SIOP-RTSG 2016 UMBRELLA protocol was launched in June 2019 [5]. It provides a standard of care for treatment (chemotherapy, surgery and radiotherapy), for all children and adults with all stages of Wilms tumor including a stratified relapse protocol and a recommendation for young infants. Moreover, it includes detailed guidance towards standard of diagnostics and treatment for children with non-WT, such as RCC, MN, CN, CPDN, MRTK and CCSK [2]. Registration of these data will improve future decision making for these rare tumors.

Pathology review according to SIOP-RTSG 2016 UMBRELLA protocol was pursued in 148/152 of the cases. Rapid pathology review allows early identification of rare tumor types and helps to adequately plan stage and histological subtype driven, risk-adapted, postoperative treatment stratification [52].

Out of 54 patients with an indication of radiotherapy, 36 patients required flank irradiation. A recent analysis of the latter group of patients provides encouraging evidence that excellent loco regional control can be obtained by combining highly conformal flank target volumes with intensity modulated arc techniques (IMRT) while sparing dose to the surrounding healthy tissue [42, 43, 53]. This is currently being implemented on a larger scale in the SIOP-RTSG 2016 UMBRELLA protocol for those countries that have facilities to apply IMRT, and international quality control settings are being generated.

National centralization has improved our ability to store data and biomaterials from the majority of our patients, after obtaining consent from patients and families. This enables the opportunity to start innovative research initiatives for pediatric renal cancer, including organoid technology, 3-D imaging, next generation sequencing and liquid biopsy analyses, which previously hardly existed in The Netherlands for these patients. Such initiatives are important to identify disease specific oncogenic drivers, cells of origin, tumor discriminators, prognostic biomarkers and molecular (novel) target identification. Moreover, this is important to boost personalized therapy development for the still incurable adverse prognostic metastatic patients. This illustrates the urgent and unmet need for translational efforts in these particular very unfortunate subsets of renal tumor patients. Such aggressive subtypes include MRTKs, diffuse anaplastic WTs, (relapsed) CCSKs, other high risk histology tumors and relapses. Our research program further includes identification of (novel) tumor predisposition syndromes and toxicity profiles and its determinants, treatment and stratification tools. This leads to the possibility of designing interventions to prevent early and late serious morbidity and mortality.

Hence, centralization of care for children with cancer led to referral of nearly all new renal tumor cases in the Netherlands. Inclusiveness of all renal tumor subtypes and a multi-disciplinary approach will further improve our insight in epidemiology as well as our understanding and expertise on the management of all renal masses. These approaches for all pediatric renal cancer patients, allowing the best available care with standard review-based diagnostics, have now been established in a national center. Further development of molecular (and other) innovation-based treatment modalities for the future will contribute to enhance early and (the quality of) long term survival.
